# Enhancing Surgical Care: Development of Biocompatible, Superabsorbent Alternatives to Cotton Gauze Using Chia Mucilage and Poly(vinylpyrrolidone)

**DOI:** 10.1021/acsomega.4c08073

**Published:** 2024-10-28

**Authors:** Kainan Akio Weege, Antônio Augusto Ulson de Souza, Andrea Cristiane Krause Bierhalz, Paulo Feuser, Ana Paula Serafini Immich

**Affiliations:** † Graduate Program in Textile Engineering, Department of Textile Engineering, Federal University of Santa Catarina, Blumenau, SC 88040-900, Brazil; ‡ Graduate Program in Chemical Engineering, Department of Chemical Engineering and Food Engineering, Federal University of Santa Catarina, Florianópolis, SC 88040-900, Brazil

## Abstract

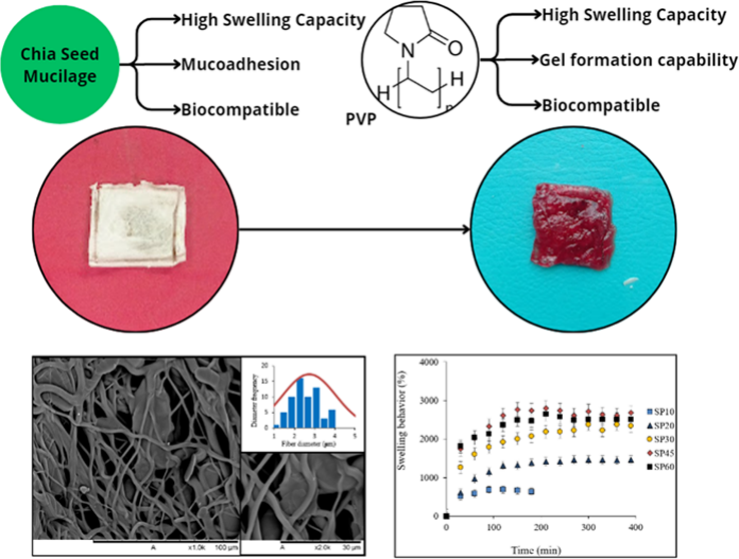

Cotton gauze bandages have traditionally played a pivotal role in wound care and surgical procedures, absorbing fluids, including blood, and protecting against infection. However, their limited liquid absorption capacity raises concern about potential post-surgery complications if inadvertently retained. In response, resorbable and biocompatible polymers have emerged as a promising alternative to enhance surgical outcomes and mitigate inflammation. This study aims to develop a biocompatible, highly absorbent, and preferably resorbable substitute for cotton gauze, utilizing natural polysaccharides from chia seeds’ mucilage alongside the synthetic polymer poly(vinylpyrrolidone) (PVP). Incorporating tranexamic acid, an antifibrinolytic agent, into the PVP solution enhances its efficacy in controlling blood flow. The polymer solution is then processed into nonwoven materials via solution blow spinning. UV–C radiation cross-linking is employed to bolster the nonwovens’ performance and durability during liquid absorption and swelling. Results demonstrate that nonwoven samples comprising PVP and chia mucilage, cross-linked for 60 min with UV–C radiation, exhibit exceptional swelling capacity, absorbing approximately 3291% of their dry weight in saline solution. Microfiber analysis indicates alterations in fiber characteristics due to cross-linking duration. Cell viability tests affirm the biocompatibility of the produced materials. With their remarkable fluid absorption properties and potential for resorption, PVP/chia mucilage compositions supplemented with tranexamic acid offer a promising avenue for effectively managing surgical bleeding without adverse effects. Furthermore, these materials can safely remain within the surgical site, eventually undergoing natural resorption by the body owing to their resorbable nature.

## 1 Introduction

Instances of foreign textile materials being inadvertently left inside patients have been documented in medical literature.^1−3^ This typically occurs during emergency procedures, lengthy surgeries, team changes, or issues related to the accurate tracking of cotton compresses. Currently, cotton gauze and compresses are the most commonly used materials for surgical interventions and general wound care, as they assist in the absorption of fluids, blood, and other secretions, in addition to being cost-effective and practical to use and store.^4,5^ The gauze has an open and loose texture, allowing fluids to be absorbed by the fibers, removed, or passed to other absorbent materials in the dressing.^6^ The primary drawback associated with this material is its limited capacity for absorbing liquids. If unintentionally left inside the body after surgery, it can give rise to a range of significant issues, such as infection, inflammation, abscess development, delayed healing, harm to organs, and patient discomfort and pain.^1,2^ Over the years, polysaccharides have been researched as a means to develop a more effective and absorbent alternative to cotton for biomedical applications.^7−9^ These pure polysaccharides have some limitations such as inadequate mechanical properties. Another alternative for cotton substitution is synthetic, hydrophilic, and biocompatible polymers such as poly(vinylpyrrolidone) (PVP).^10^ Poly(vinylpyrrolidone) (PVP) nanofibers show great potential in biomedical applications due to their high surface area, porosity, and biocompatibility. The solution blow spinning technique stands out as a modern method for producing these nanofibers, surpassing traditional electrospinning in terms of production rate, uniformity, and material versatility. These nanofibers are ideal for drug delivery systems, wound dressings, filtration, and tissue engineering, with the potential to expand their use in healthcare and environmental solutions.^11,12^ To enhance the structure, a synthetic polymer was added, which is soluble in water and ethanol, that can be cross-linked through UV–C light irradiation,^13^ producing a hydrogel capable of absorbing 25 g/g of its dry weight in water.^14^ In addition to its biocompatibility, it can simultaneously swell and release drugs for local treatment.^15^ To increase its absorption, mucilaginous agents such as those present in chia can be used since they have interesting moisture absorption properties, swelling up to 27 g/g of their dry weight in water.^16^ However, there are no reports in the literature about the capacity of mucilages to absorb blood. Tranexamic acid (ATX), a synthetic derivative of the amino acid lysine, has been chosen to be incorporated into the manufactured structure to aid in blood clotting.^17^

This work aims to develop a biocompatible and bioresorbable nonwoven biotextile as an alternative to cotton gauze. In addition to PVP presenting good biocompatibility, studies have shown that chia mucilage also presented good degradability. A study on chia mucilage degradation examines in vitro enzymatic breakdown, emphasizing its potential in diverse applications, including cosmetics and packaging. Results showed an initial mass increase after 24 h due to polymer swelling, followed by a mass loss of 6.34%. Over time, this loss escalated to 49.19% at 72 h and reached 83.17% at 168 h.^18^ This material, produced via solution blow spinning (SBS) using PVP as the primary matrix and chia mucilage as an augmentation, seeks to enhance moisture absorption, reduce blood flow, and promote healing in surgeries. Emphasizing improved swelling properties, this absorbable material could serve as a surgical compress, offering patients faster recovery with minimal discomfort during the postoperative period.

## 2 Materials and Methods

**Figure 1:**
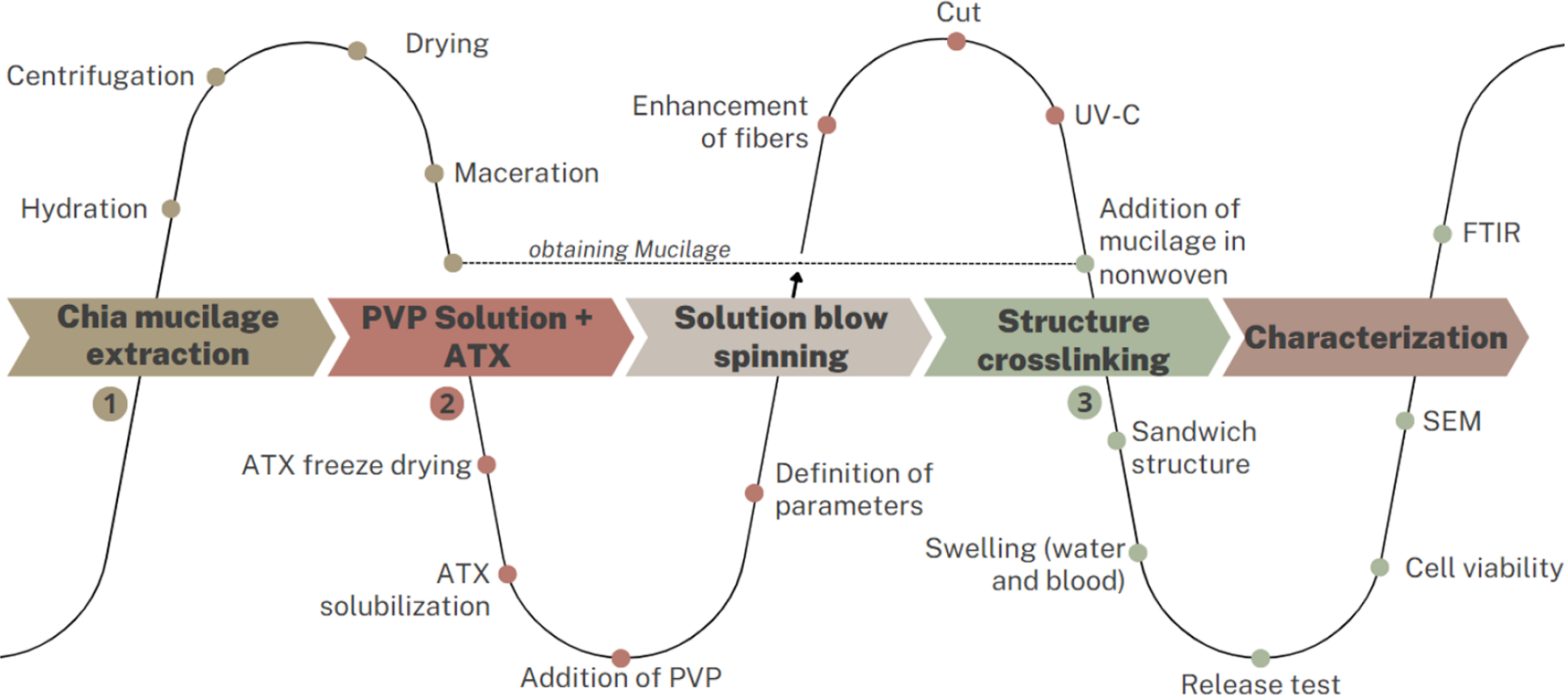
Overall flowchart of the experimental methodology.

For the production of biotextile, the following materials were used: Poly(vinylpyrrolidone) (PVP - K90) (Mw 360,000 g/mol) from Fluka brand (supplier Sigma-Aldrich) and chia (*Salvia Hispanica L*.) obtained from a natural products store (Grãos e Cia - Produtos Naturais) located in Blumenau, Brazil. Tranexamic acid (Transamin) was obtained from a local hospital with a concentration of 50 mg/mL (Mw 157.2 g/mol). The solvent used was absolute ethyl alcohol 99.8% P.A (Mw 46.07 g/mol) from NEON supplier. For the swelling analysis, physiological saline, obtained from a local pharmacy, as well as fresh porcine blood, obtained from Glau Defumados LTDA (Slaughterhouse located in Blumenau, Brazil), were used. Figure 1 shows the steps carried out in this study.

### 2.1 Extraction of Chia Mucilage

The extraction of chia mucilage was carried out according to the methodology described by Dick et al.^19^ For mucilage extraction, chia seeds were hydrated in water at a ratio of 1:40 w/w at 25 °C and mixed on a magnetic stirrer for 2 h.

To better extract the mucilage from the seeds, a BMX201 mixer (Britânia) was used with the blades covered with tape to avoid crushing the seeds. This process helps to separate the mucilage from the chia seeds, which are extremely adhesive to the seeds. The extract was separated from the seeds by centrifugation at 400 rpm for 5 min. To remove the seeds not precipitated in this process, the mucilage was filtered using a simple filter. After this stage, the mucilage was dried in an oven at 50 °C for 12 h. The final mucilage obtained was ground into a fine powder with the aid of a mortar and pestle and passed through a 100-μm mesh.

#### 2.1.1 Mucilage Extraction Yield

The percentage yield of chia mucilage extraction was determined in duplicate as the ratio between the dry mass of powdered mucilage and the mass of the seed according to eq 1.
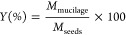
1where, *Y* (%) is the extraction yield, *M*_mucilage_ is the dry mass of mucilage, and *M*_seeds_ is the dry mass of the used seeds.

### 2.2 Drug Lyophilization

The purpose of lyophilization is to remove water from the drug to facilitate spinning with PVP. Tranexamic acid (ATX), in 5 mL ampules at a concentration of 50 mg/mL, was lyophilized for 24 h (Liotop brand, LD101 model at a temperature of −55 °C under a pressure of 40 μm·Hg). The lyophilized drug was stored in a desiccator until use.

### 2.3 Preparation of PVP Solution

Initially, the lyophilized ATX was dissolved in anhydrous ethanol at a concentration of 10 mg/mL under magnetic stirring at room temperature. After the ATX was dissolved, PVP was slowly added to this solution at a concentration of 10% w/v maintaining the stirring for 2 h.

### 2.4 Solution Blow Spinning Equipment Setup

**Figure 2:**
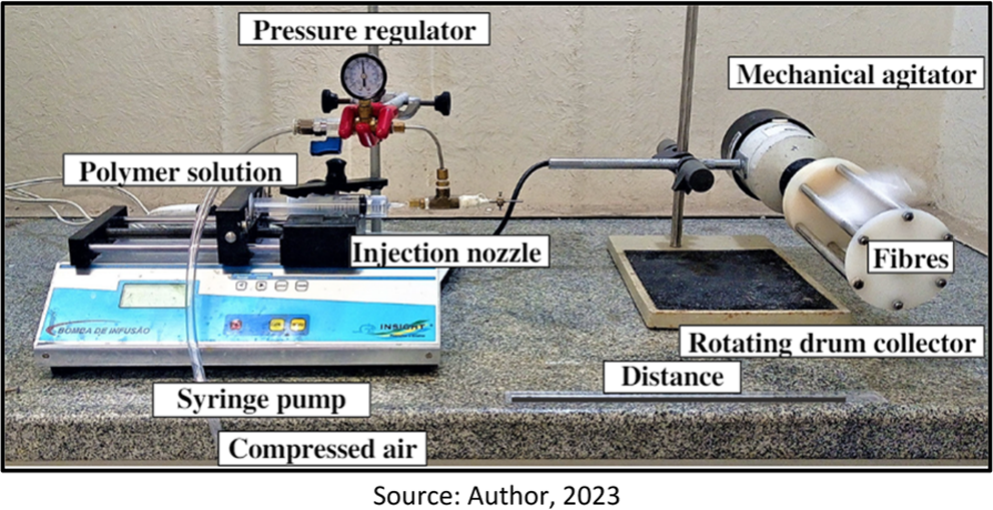
Solution blow spinning equipment.

The solution blow spinning technique has a basic configuration consisting of an air compressor, a pressure regulator, an infusion pump that pull the polymer solution in to the injection nozzle, a metallic extrusion nozzle, a mechanical agitator, and a rotating drum collector, as shown in Figure 2.

The polymeric solution was spun in a 20 mL syringe with a 12.5 cm length and a nozzle for easy connection to the metallic extrusion nozzle of the equipment. For this process, a cylindrical metal collector connected to a mechanical stirrer was also used. For the inner nozzle, tubes with an outside diameter (OD) of 1.6 mm and an inside diameter (ID) of 1.0 mm were used. The protrusion used was 1.5 mm, 30 cm away from the collector, with an injection of 42 μL/min with an air pressure of 3 bar.

### 2.5 Cross-Linking of the Nonwoven by UV–C Radiation

As the polymer used in the production of the nonwovens is hydrophilic, a cross-linking process was employed to impart appropriate resistance in liquid media such as sweat or blood. For cross-linking, the produced samples with 1.5 cm × 1.5 cm were irradiated with UV–C light with a wavelength of 254 nm for 10, 20, 30, 45, and 60 min on both sides of the structure. This procedure was carried out in a radiation chamber prototype developed by the Mass Transfer Laboratory of the Department of Chemical Engineering and Food Engineering at the Federal University of Santa Catarina.

### 2.6 Sandwich Structure Configuration

The sandwich configuration was formed by adding chia mucilage between two layers of PVP and PVP-ATX membranes previously obtained by the SBS process. The PVP layers were made up of 2.25 cm^2^ samples and the chia mucilage was added at 30% chia mucilage to the total mass of PVP, forming a 3D microfiber structure. This structure was sealed at the edges with the aid of a heated metal surface to store and protect the mucilage between the layers of PVP.

### 2.7 Swelling Capacity Test

The samples were immersed in solutions of physiological serum and fresh porcine blood at room temperature and were weighed at 30 min intervals to monitor the degree of swelling. The sample with 60 min of cross-linking was chosen to carry out the blood tests. The tests were performed in triplicate. The degree of swelling (SD) was calculated according to eq 2, where *M*_f_ is the stabilized mass in the aqueous medium and *M*_i_ is the mass obtained initially before immersion in the solutions used.
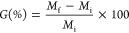
2

### 2.8 Scanning Electron Microscopy (SEM)

The morphology of the obtained microfibers was evaluated through a Hitachi scanning electron microscope, model TM3030. The fibers were previously coated with a nanometer-thick layer of gold. The measurement of the average diameters of the obtained fibers was performed with the aid of ImageJ software, version 1.53. The histograms were obtained from the mean of 75 fiber diameter measurements. The samples analyzed by SEM were uncross-linked PVP, PVP cross-linked for 20 min with UV–C, and PVP cross-linked for 60 min with UV–C.

### 2.9 Cell Viability Analysis—MTT

In order to assess the cell viability of the produced nonwovens, the MTT (3-[4,5-dimethylthiazol-2-yl]-2,5 diphenyl tetrazolium bromide) assay was performed. The evaluated samples were pure cross-linked PVP spun via the SBS technique, cross-linked PVP containing ATX spun via the SBS technique, and just chia mucilage.

Cell viability of the different samples was evaluated in a murine fibroblast cell line (NIH3T3). The cells were cultured in Modified Dulbecco’s Eagle Medium (DMEM) supplemented with 10% fetal bovine serum, penicillin (100 units/mL), streptomycin (100 mg/mL), and 4 mM/L glutamine at 37 °C in 25 mL cell culture bottles and maintained in an incubator at 37 °C with 5% CO_2_. All reagents were obtained from Sigma-Aldrich. For experimental purposes, cells were trypsinized and adjusted to a concentration of 104 cells/well and plated in a 96-well flat-bottom culture plate. After the incubation time (24 h), the cells were treated with DMEM medium containing the different samples (PVP, PVP + drug, chia) solubilized in DMSO (pure extract) and PBS, and then were incubated again at 37 °C with 5% CO_2_ for 24 h. After this time, the cells were washed with PBS, and cell viability was measured using an MTT assay. In each well, a mixture of 20 μL of the MTT reagent and 100 μL of the medium was added, and the plate was incubated again at 37 °C for 2 h with 5% CO_2_. Subsequently, the remaining medium was transferred to 96-well plates for measurements at a wavelength of 490 nm. The analyses were performed by using a spectrophotometer (Molecular Devices, Spectra Max Plus 348) with four parallel replicates for each sample. The culture medium was used as the control group.

### 2.10 Hemolysis Assay

All experiments were conducted following the Ethics Research Committee (CEP) of the University of Extremo Sul Catarinense, which approved them before the start of experimental procedures under authorization number 3344689. Human erythrocytes were obtained from three healthy volunteers. The whole blood, 3 mL, was centrifuged at 1500 rpm for 30 min, three times, to remove other cells and traces of plasma. After the third wash, the supernatant was discarded and the erythrocytes were resuspended in 3 mL of saline solution (0.9% NaCl).

After the erythrocytes were obtained, hemolytic activity was assessed by spectrophotometry. The selected compounds for this assay were incubated at different concentrations (0.1, 0.5, and 1 mg/mL), containing 50 μL of resuspended human erythrocytes in a final volume of 1 mL of a saline solution. The incubation time was 1 h at 37 °C under continuous agitation at 100 rpm. After the incubation period (1 h), the solution was centrifuged for five min (10,000 rpm) at room temperature. Subsequently, 100 μL of the supernatant from the sample was transferred to a 96-well plate. The percentage of hemolysis was assessed by spectrophotometry. Additionally, the absorbance was measured at 540 nm using an Infanite 200 TECAN microplate reader. Positive control (distilled water) and negative control (saline solution) were also performed. The results were expressed as the percentage of hemolysis obtained from eq 3 below:
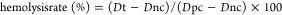
3where, *D*t = absorbance of the test sample; *D*nc = absorbance of the negative control; *D*pc = absorbance of the positive control. The assays were performed in triplicate.

## 3 Results and Discussion

### 3.1 Mucilage Extraction

**Figure 3:**
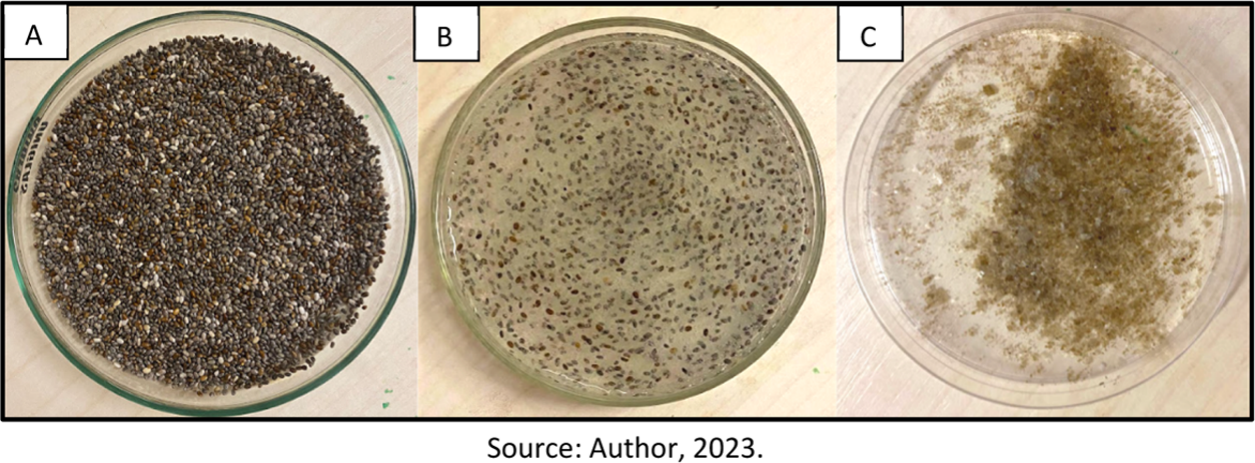
Mucilage phases during the extraction process. (A) Chia seeds; (B) Mucilage released from seeds; (C) Mucilage obtained after drying process.

The yield of extracted chia mucilage, calculated according to eq 1, was 6%. This value is similar to those reported by Muñoz et al.,^16^ who obtained a yield of 6.97%. It is emphasized that mucilage yields are primarily affected by the extraction techniques employed, particularly the application of the temperature during the hydration phase, different hydration times, different seed/water ratios, and drying methods such as oven drying or freeze-drying. Figure 3 shows the visual appearance of the chia seeds (6A), the mucilage detached from the hydrated seeds (6B), and the mucilage obtained after extraction and drying (6C).

During the hydration stage, the mucilage had a colorless appearance, which was induced by a high water concentration. The final mucilage appeared as a beige powder with a characteristic sweet odor and had a thin film-like structure, resembling scales.

### 3.2 Solution Blow Spinning of PVP Solution

To find the optimal spinning conditions, the influence of solution parameters (concentration) and process parameters (distance and pressure) was evaluated. An important parameter for the SBS technique, such as protrusion, was set at 1.5 mm based on previous studies.^20^ For the inner nozzles, tubes with an outside diameter (OD) of 1.6 mm and an inside diameter (ID) of 1.0 mm were used. These parameters were adjusted based on fiber production on the collector, considering the ease of production, the quantity of fibers produced, and their quality.

**Figure 4:**
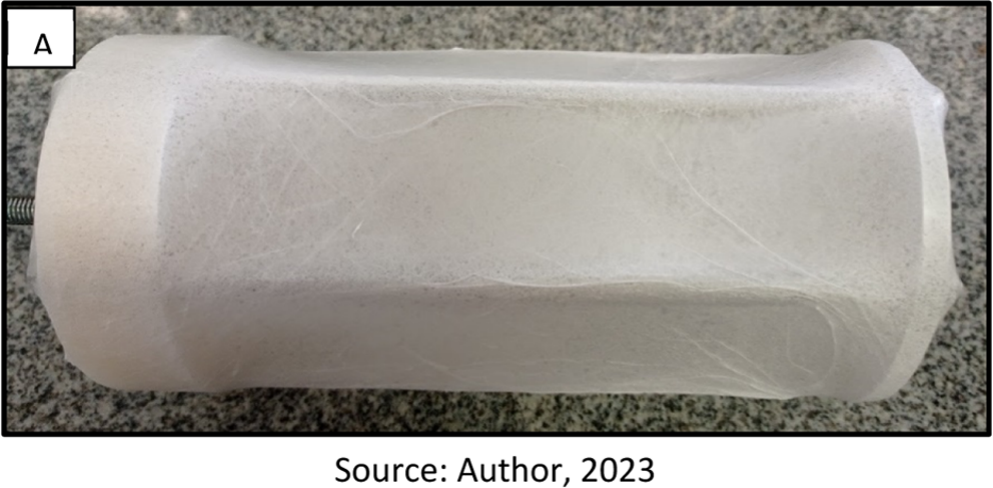
PVP fiber was obtained by solution blow spinning after adjustment of the parameters.

In Figure 4 are shown PVP fibers produced with adjusted parameters such as polymer concentration (10%), collector distance (30 cm), and air pressure (4 bar). This structure exhibited a uniform appearance with the presence of a tangle of strong and consistent fibers that did not easily coalesce when handled compared to the fibers produced without the adjusted parameters. Scanning electron microscopy images will be presented in the characterization section to validate the obtained diameter distribution.

Following the fabrication of the PVP nonwovens, the samples underwent UV–C irradiation for specified time intervals (10, 20, 30, 45, and 60 min) to induce cross-linking within the polymer matrix and alter its solubility in aqueous environments, such as saline or blood.

### 3.3 Calculation of the Gel Fraction of Cross-Linked Nonwovens

**Figure 5:**
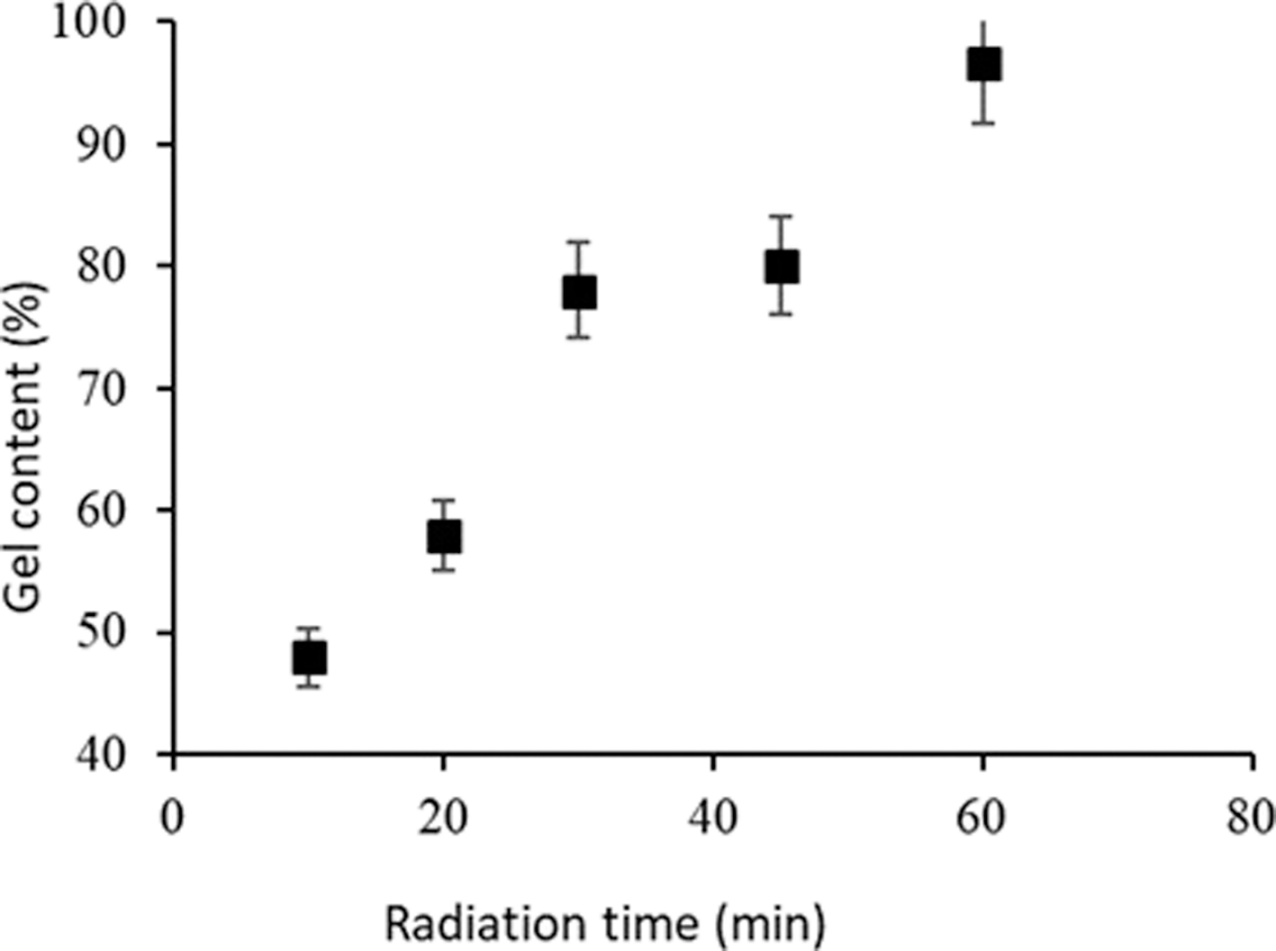
Sol–gel fraction of PVP nonwoven for all cross-linking times (10, 20, 30, 45, and 60 min).

To verify the influence of UV–C radiation cross-linking on the stability of nonwovens in an aqueous environment, the gel fraction was calculated. The gel fraction represents the cross-linked portion of the polymer that became insoluble, while the sol fraction is the portion that dissolved in an aqueous solution. Figure 5 presents the gel–sol fractions of the fibers produced with pure PVP (SP) for all studied cross-linking times.

Results indicated that the longer the exposure time to UV–C radiation, the higher the gel fraction of the PVP sample. The determined gel fraction of the nonwoven samples exhibited a growth from 48% to 96% as the fibers underwent UV–C irradiation for periods of 10 and 60 min, respectively. The increase in gel fraction implies higher stability of the samples in aqueous media. This attribute can be ascribed to the development of cross-links within the PVP polymer matrix, which transpire upon exposure to UV–C radiation, consequently altering the polymer’s molecular chains. The gel fraction of the non-cross-linked polymer could not be determined as the polymer entirely dissolved in an aqueous solution. Immich (2009)^21^ confirms that the longer the exposure time to UV–C radiation, the higher the gel fraction of the formed polymer. For exposure times longer than 30 min, UV–C radiation already substantially modifies the polymer chains, enabling the nonwoven to trap liquids in its structure without degrading for several hours.

### 3.4 Scanning Electron Microscopy (SEM)

**Figure 6:**
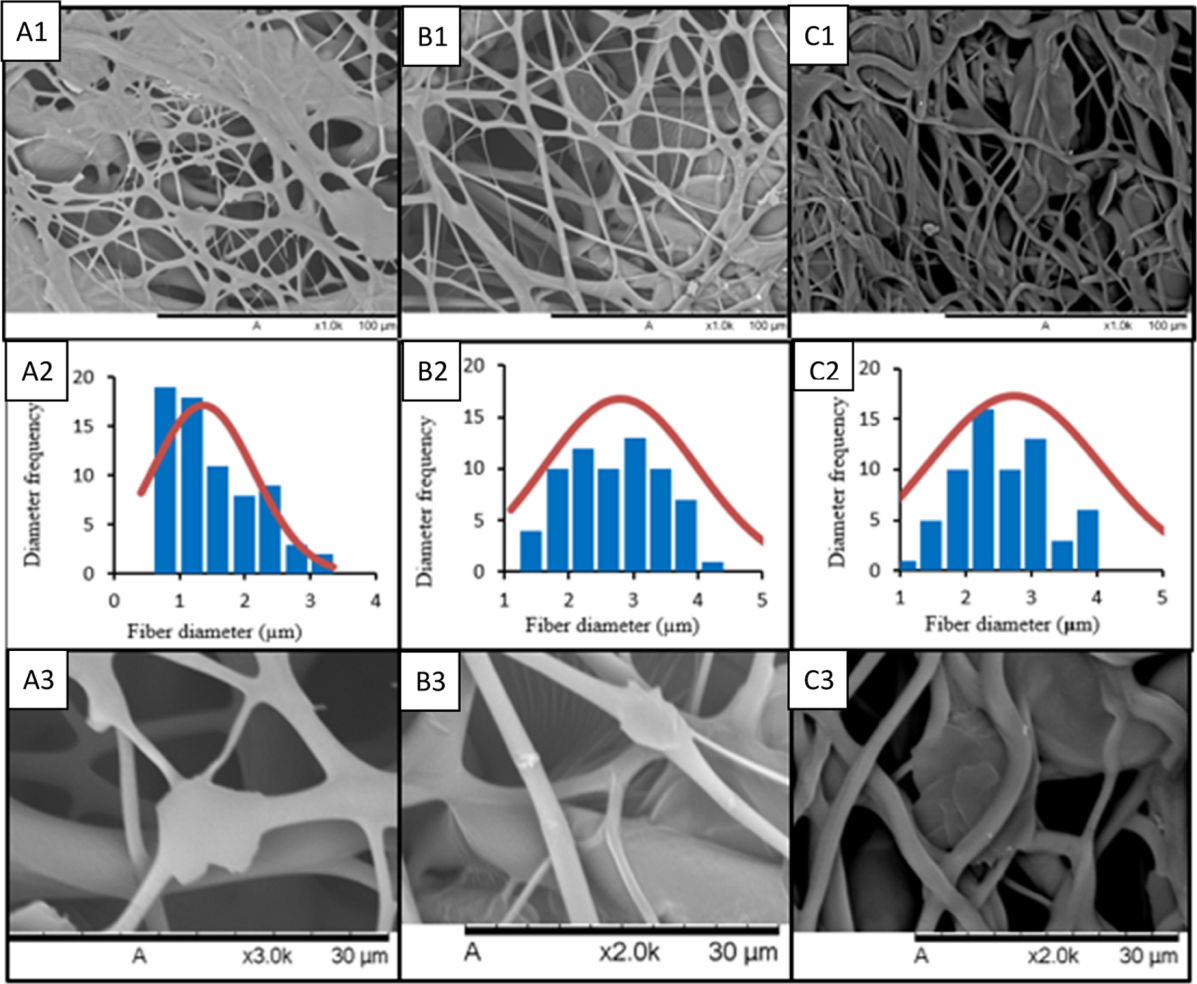
[A] SEM for nonwoven before cross-linking; (A1) 1000× magnification; (A2) fiber diameter distribution frequency for nonwoven; (A3) 3000× magnification. [B] SEM for nonwovens after 20 min of cross-linking; (B1) 1000× magnification; (B2) fiber diameter distribution frequency for nonwoven; (B3) magnification of 3000×. [C] SEM for nonwovens after 60 min of cross-linking; (C1) 1000× magnification; (C2) fiber diameter distribution frequency for nonwoven; (C3) magnification of 3000×.

The morphology of the produced nonwovens was evaluated before cross-linking and after cross-linking times of 20 and 60 min. The cross-linking times of 20 and 60 min were chosen to represent short and long cross-linking times, respectively. Figure 6 presents SEM images of the non-cross-linked nonwovens and the nonwovens cross-linked with 20 and 60 min, along with the fiber diameter distribution graph.

The [A] nonwoven before cross-linking reveals the formation of porous structures with interconnected pores. However, the fibers are dense, which can enhance their strength and mechanical properties. The fibers exhibited an average diameter of 1.358 ± 0.631 μm. The minimum and maximum values found were 0.386 and 3.336 μm, respectively, indicating the production of fibers in the micron range. These results are similar to those reported by Cena et al.^12^ where PVP fibers (with the same molecular weight) produced by SBS had average diameters ranging from 0.75 to 1.25 μm.

The [B] nonwoven after 20 min of cross-linking presents SEM images along with the fiber diameter distribution graph. The average fiber diameter was found to be 2.797 ± 0.821 μm. The formation of beads was not observed, suggesting that the polymer concentration used was suitable for the spinning process and solvent evaporation was efficient. Bright spots on the fiber surface may be due to the gold coating, which was applied to improve the conductivity and enhance fiber visualization.

According to Oliveira et al.,^22^ in the SBS process, three factors directly contribute to obtaining fibers with nanoscale morphology: solution concentration, pressure, and injection rate. Both analyzed nonwovens exhibited fiber diameters ranging from approximately 1 to 4 μm, confirming that SBS produces fibers with larger diameters compared to electrospinning. This finding is supported by the results of Asawahame et al.^23^ and Cena et al.,^12^ who obtained PVP fibers with diameters ranging from 0.55 to 0.95 μm and 0.75 to 1.25 μm, respectively, for the same PVP concentration (10%). Slightly larger fiber diameters were observed by Gao et al. (2020),^24^ who obtained fibers with an average diameter of 2.21 ± 0.884 μm.

The [C] nonwoven after 60 min of cross-linking, the fibers appear tangled, forming a network of microfibers that are interconnected. With the cross-linking process using UV–C radiation, the fibers exhibited slightly larger diameters and a more flattened or twisted shape. The average diameter obtained was 2.744 ± 0.884 μm. The morphological analysis of the nonwovens, before and after cross-linking, reveals the fibrous and porous structure formed using the solution blow spinning technique. This analysis does not indicate any signs of damage or degradation to the nonwoven structure after 60 min of cross-linking. Signs of structural damage include brittle fibers, fiber discontinuity, and the presence of residues.

### 3.5 Swelling of PVP Nonwovens in Physiological Saline Solution

The swelling of the PVP nonwovens was measured to evaluate the water retention capacity of the hydrophilic and cross-linked polymer nonwoven structure. Initially, swelling tests were performed for PVP nonwovens in physiological saline solution, for exposure times of 10, 20, 30, 45, and 60 min to UV–C radiation.

**Figure 7:**
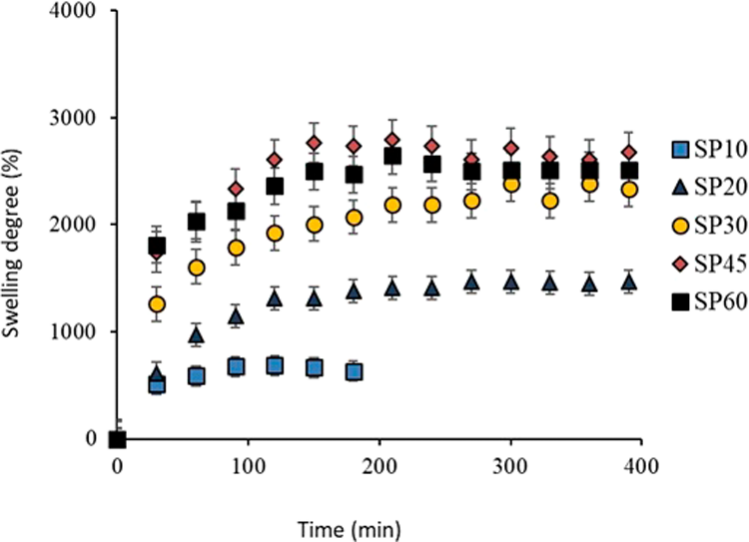
Degree of swelling of pure PVP for times of 10, 20, 30, 45, and 60 min of cross-linking in PBS (0,01 M).

According to Burkert et al.,^25^ the degree of swelling of cross-linked polymeric materials depends on the cross-linking density of the polymer system. Additionally, swelling is also influenced by the thickness of the PVP layer, the mass of mucilage, and the porosity of the fabricated structure. To estimate the influence of swelling in the sandwich structure (PVP + mucilage), the degree of swelling was initially calculated for sandwiches produced with pure PVP (SP), as shown in Figure 7, for all studied cross-linking times.

The findings indicate that the cross-linked structure exhibited excellent ability to retain physiological saline solution, which became apparent within the initial 30 min of the swelling test. Notably, a substantial improvement was observed after a 60 min cross-linking process. Throughout the experiment, the nonwoven materials consistently displayed a gel-like structure and progressively absorbed more solution over time. As for the non-cross-linked nonwovens, their results were not presented due to their rapid dissolution in the solution, rendering a thorough swelling analysis unfeasible.

The nonwoven exposed to UV–C for 10 min showed partial solubilization of the structure, possibly due to the low gel fraction formed by cross-linking (48% gel fraction, as shown in Figure 7). The highest swelling was achieved for the nonwoven exposed to UV–C for 45 min, swelling approximately 2800% within a period of 3.5 h. According to Ajji, Othman, and Rosiak,^26^ in the initial stage of the curve, the expansion rate is very high (up to 2 h) and the fluid can easily penetrate the polymer network, mainly due to the high availability of empty spaces between the previously cross-linked chains, facilitating fluid penetration into the nonwoven.

The swelling observed in the sample exposed to 60 min of UV–C irradiation was less pronounced compared to the sample exposed to 45 min of UV–C light. This difference may be attributed to the simultaneous occurrence of polymer chain breakdown and cross-link formation during the cross-linking process. Excessive UV–C radiation has the potential to trigger the depolymerization of PVP, thereby modifying both the mechanical properties and swelling characteristics of the nonwoven material.^21^

### 3.6 Swelling of PVP and Chia Mucilage Sandwiches (SPM) in Porcine Blood

**Figure 8:**
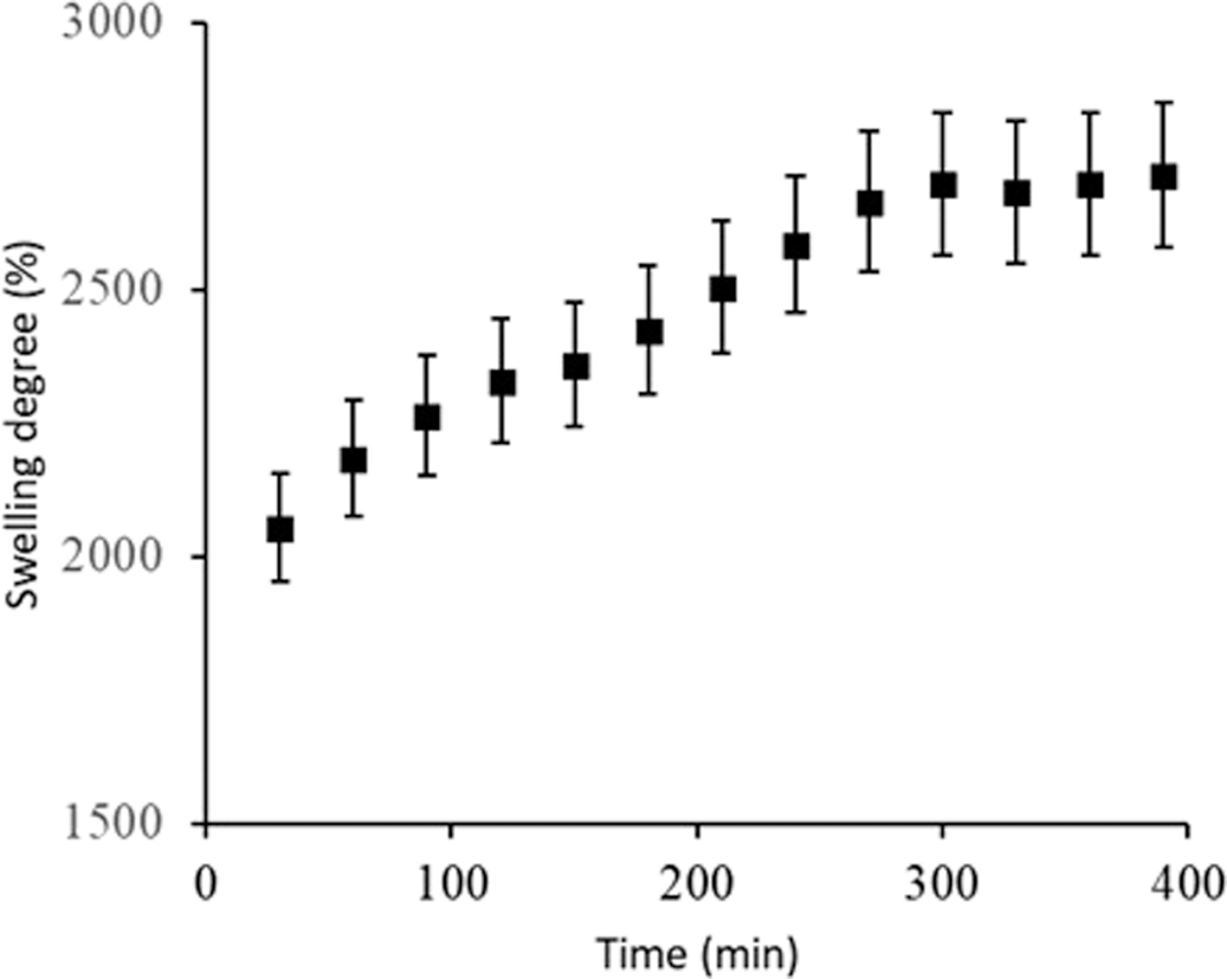
Degree of swelling of PVP and chia mucilage in porcine blood.

The swelling results of the sandwiches in porcine blood are presented in Figure 8. An accelerated swelling is observed in the first 30 min of the experiment, where the sandwich absorbed a considerable amount of blood, swelling by approximately 2000%. This initial absorption-driven swelling could be attributed to the large surface area of the PVP microfibers. After this accelerated swelling, the sandwich continues to absorb at a slower rate, reaching its maximum absorption capacity around 5 h of testing, with a swelling degree of 2712%.

The blood absorption capacity of the sandwich structure was lower compared to the results obtained for absorption in physiological saline, which may be related to the different constituents present in the blood. Blood is composed of a liquid component (plasma) consisting of water, salts, vitamins, and coagulation factors, in which solid components such as red blood cells, leukocytes, and platelets are suspended. According to Shahrabi, Barzin, and Shokrollahi,^27^ the hydrophilicity of PVP hydrogels improves the permeation of red blood cells and platelets. However, the authors indicate that high concentrations (10–20%) of PVP can negatively influence the permeation of red blood cells and platelets within the structure as there is a wide distribution of fibers and small pores that consequently increase flow resistance. This also affects the swelling rate as the permeation time is proportional to the increase in PVP concentration.

**Figure 9:**
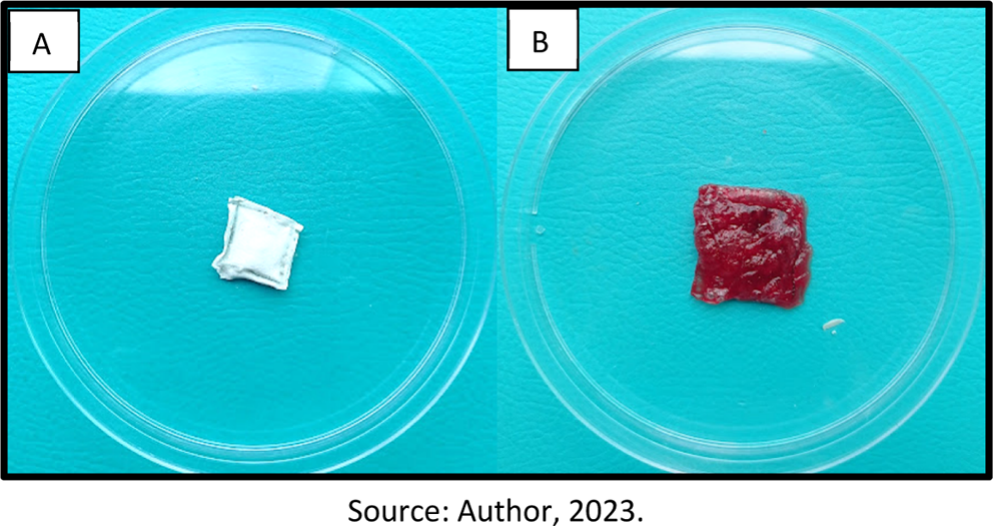
Visual appearance of sandwiches made with PVP and chia mucilage, swollen in porcine blood. (A) Before swelling. (B) After swelling.

Figure 9 presents the appearance of the swollen nonwovens with porcine blood. The SPM60 sandwich exhibited a characteristic reddish appearance due to the blood components, such as hemoglobin. Its structure also did not solubilize or release the mucilage during the analysis period, indicating that the PVP coating was sufficient to adequately encapsulate the mucilage.

In the image presented in Figure 9, the increased size of the sandwich structure due to swelling can be visualized. The integrity of the structure is also evident as it retains all of its mucilaginous content within the sandwich.

### 3.7 Cell Viability Assay

**Figure 10:**
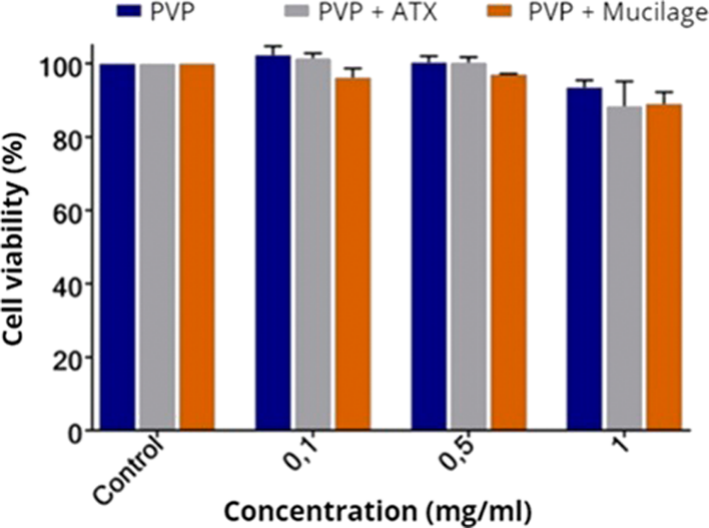
In vitro cytotoxicity assay of different concentrations of pure PVP, PVP + ATX, and chia mucilage. Significant differences are shown (*p* < 0.05 when compared to the control group—two-way ANOVA followed by Tukey’s test).

The cell viability of pure PVP fibers, PVP + ATX, and chia mucilage sandwiches samples was evaluated in murine fibroblast cell line (NIH3T3). The cytotoxic effect was assessed, and the results did not indicate a reduction in cell viability (*p* < 0.05) at concentrations ranging from 0.1 to 1 mg/mL. Figure 10 presents the result of the in vitro cell viability assay conducted for different concentrations of pure PVP, PVP + ATXg, and chia mucilage.

As shown in Figure 10, no sample exhibited cytotoxic effects at different concentrations, indicating cell viability higher than 90% for the more concentrated samples.

**Figure 11:**
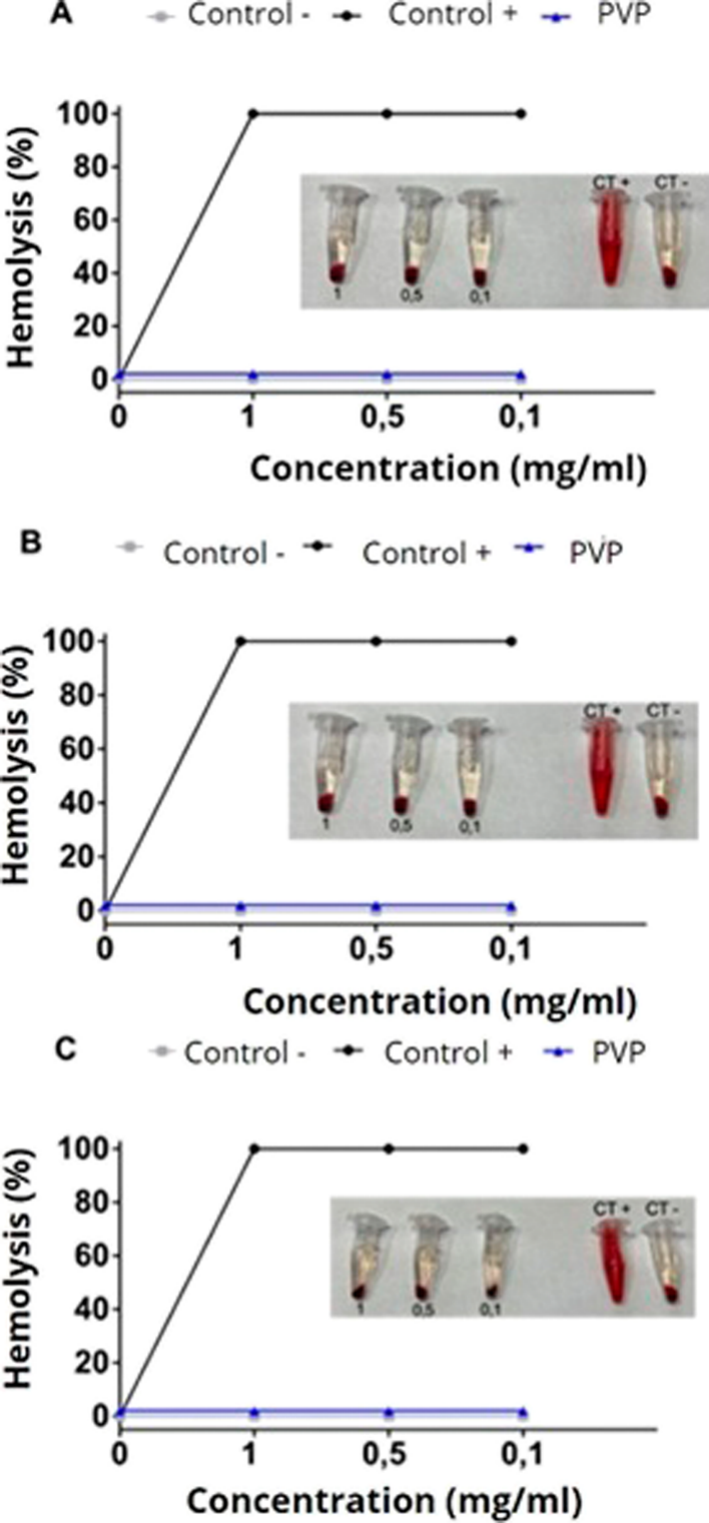
Hemolysis test of the samples: (A) pure PVP; (B) PVP + ATX; and (C) chia mucilage. Significant differences are shown (*p* < 0.05 when compared to the control group—two-way ANOVA followed by Tukey’s test).

These results are further supported by the hemolysis assays. The hemolysis assay is important for evaluating cytotoxicity specifically for red blood cells (erythrocytes). According to the criteria of the Standard Test Method for Analysis of Hemolytic Properties of Nanoparticles (ASTM E2524-08), a hemolysis percentage >5% indicates that the tested material may cause damage to cells. Figure 11 presents the results of the hemolysis assay.

According to the data presented in Figure 11, the percentage of hemolysis for the tested samples is significantly below 5%, indicating a safe hemolysis percentage for the application of the produced biotextile, as stated in ISO/TR 7406.24. This indicates the nontoxicity of the biomaterial. These results are consistent with the cell viability results for fibroblasts. Therefore, it can be concluded that the PVP nanofibers with drug and chia, cross-linked for 60 min using UV–C radiation, do not exhibit cytotoxic characteristics and can serve as an alternative to replace cotton gauzes in surgical applications for blood containment.

## 4 Conclusions

In this study, solution blow spinning (SBS) was used to create a novel biotextile: a fibrous nonwoven designed as a PVP/chia mucilage sandwich with tranexamic acid (ATX) for potential biomedical applications. Functionally, this material demonstrated good properties, with resulting PVP hydrogels proving entirely nontoxic and boasting impressive gel formation and swelling capabilities. Our nonwoven showcased dense, intact microfibers, with average diameters suitable for easy permeation by blood components. Notably, the structure cross-linked for 60 min exhibited superior stability and absorbency in physiological saline. With its remarkable biocompatibility and fluid absorption capacity, our biotextile holds promise for surgical bleeding control without adverse effects. The resorbable nature of the materials used further enhances their potential, allowing for safe retention within the injury site and eventual absorption by the body. In conclusion, our findings highlight the SBS technique’s potential for rapid production of microfibrous nonwovens, offering a promising avenue for impactful biomedical applications in surgical care
